# Genomic Insights into *bla*_NDM-5_-Producing *Escherichia coli* ST648 Isolates from Human and Wildlife Sources in Lebanon

**DOI:** 10.3390/microorganisms13122824

**Published:** 2025-12-11

**Authors:** Zahraa F. Samadi, Ziad C. Jabbour, Zeinab R. Hodroj, Hadi M. Hussein, Abdallah Kurdi, Lama Hamadeh, Rami Mahfouz, Mahmoud I. Khalil, Rana El Hajj, Ghassan M. Matar, Antoine G. Abou Fayad

**Affiliations:** 1 Department of Experimental Pathology, Immunology and Microbiology, Faculty of Medicine, American University of Beirut, Beirut 1107 2020, Lebanonzrh02@mail.aub.edu (Z.R.H.);; 2 Center for Infectious Diseases Research, American University of Beirut, Beirut 1107 2020, Lebanon; 3 World Health Organization (WHO) Collaborating Center for Reference and Research on Bacterial Pathogens, Beirut 1107 2020, Lebanon; 4 Department of Biological Sciences, Faculty of Science, Beirut Arab University, Beirut 1107 2809, Lebanon; 5 Department of Biochemistry and Molecular Genetics, Faculty of Medicine, American University of Beirut, Beirut 1107 2020, Lebanon; 6 Department of Pathology and Laboratory Medicine, American University of Beirut Medical Center, Beirut 1107 2020, Lebanon; 7 Pillar Genomics Laboratory, American University of Beirut, Beirut 1107 2020, Lebanon; 8 Molecular Biology Unit, Department of Zoology, Faculty of Science, Alexandria University, Alexandria 21568, Egypt

**Keywords:** *Escherichia coli*, carbapenem-resistant, New Delhi metallo-beta-lactamase, ST648, whole-genome sequencing

## Abstract

*Escherichia coli* sequence type 648 (ST648), a lineage within the clinically important phylogroup F, has disseminated worldwide in humans and animals. In this study, we performed whole-genome sequencing and comparative genomic analysis for two New Delhi metallo-beta-lactamase (*bla*_NDM-5_) carrying *E. coli* strains: ECsOL198, recovered from a wild Eurasian otter in Northern Lebanon, and ECOL247, isolated from a hospitalized leukemia patient. Both isolates belonged to phylogroup F and serotype O9:H4, and exhibited IncFIA, IncFIB, and IncFII plasmids. They shared a similar antimicrobial resistance profile, including a carbapenemase gene (*bla*_NDM-5_), β-lactamase genes (*bla*_TEM-1_, *bla*_CTX-M-15_, and *bla*_OXA-1_), and other genes that confer resistance to aminoglycosides (*acc(3)-Ile*, *aadA2*), sulfonamides (*sul1*), tetracyclines (*tet*(A)), and fluoroquinolones (mutations in *gyrA* and *parC*). Both isolates also carried common virulence-associated genes related to adhesion, iron acquisition, environmental persistence, and immune evasion. Whole-genome multilocus sequence typing (wgMLST) revealed that both isolates formed a distinct subclade closely related to a bloodstream-derived ST648 isolate from India, indicating limited relatedness to global clones. These findings highlight the transmission of nearly clonal multidrug-resistant *E. coli* ST648 in both clinical and non-clinical settings, raising concerns about the threat to public health.

## 1. Introduction

*Escherichia coli* (*E. coli*) is an important Gram-negative bacterium that commonly inhabits the gut of humans and warm-blooded animals. Although most *E. coli* strains are considered harmless, others can potentially cause serious enteric and extra-intestinal infections in both humans and animals [1]. Among these, *E. coli* ST648 is reported globally as an emerging resistance-associated lineage that occurs as a pathogen and commensal in humans and animals and in the environment [2]. *E. coli* is highly clonal, as it is recognized with eight major phylogroups (A, B1, B2, C, D, E, F, and clade I) that comprise several unique sequence types (ST) detected by multilocus sequence typing (MLST) [3].

ST648 clone is recognized for its high pathogenic potential, due to its frequent carriage of antimicrobial resistance and virulence genes. Strains of this lineage often harbor resistance determinants to critical antibiotics, such as extended-spectrum β-lactamases (ESBLs) like *bla*_CTX-M_, carbapenemases such as *bla*_NDM_, and the colistin resistance gene *mcr-1*, posing a significant public health threat. Clinical isolates of ST648 are typically extraintestinal pathogenic *E. coli* (ExPEC) and are associated with bacteremia, respiratory, urinary, and wound infections. The global burden of antimicrobial resistance highlights *E. coli*, including ST648, among the leading causes of death due to antibiotic-resistant infections [4].

Antimicrobial resistance (AMR) in ST648 includes resistance to fluoroquinolones, third-generation cephalosporins, carbapenems, and other antibiotics [5]. Over the last decade, carbapenems have been recognized as last-resort antibiotics for the treatment of severe bacterial infections caused by multidrug-resistant (MDR) bacteria [6]. The emergence of carbapenem-resistant *E. coli* (CREC) has increased rapidly over the last decades and significantly limited therapeutic options [7]. One of the most clinically significant carbapenemases is the New Delhi metallo-β-lactamase (NDM), which has shown rapid evolution and global dissemination [8]. Among NDM variants, *bla*_NDM-5_ is particularly concerning due to its enhanced carbapenem resistance, attributed to two amino acid substitutions (Val88Leu and Met154Leu) [9]. It was first identified in 2011 in the MDR *E. coli* ST648 isolate in the United Kingdom [10]. Since then, NDM-producing ST648 *E. coli* strains have been reported in several countries, including Japan [11], Algeria [12], the United Kingdom, and India [13].

*bla*_NDM-5_ is mostly associated with international high-risk *E. coli* clones, such as ST167, ST361, ST405, ST410, and ST648, which cause extraintestinal and nosocomial infections in humans [14,15,16]. Notably, the first report of *E. coli* ST648 and ST156 co-producing *bla*_NDM-5_ and *mcr-1* was from a single Muscovy duck (*Cairina moschata*) [17]. Furthermore, a 2019 study from China identified the presence of *bla*_NDM-5_-producing *E. coli* in migratory birds [18]. Despite the scarcity of data on carbapenemase-producing *E. coli* in wildlife, this sector has been increasingly recognized as an important natural reservoir and disseminator of AMR. The aim of this study was to characterize multidrug-resistant, *bla*_NDM-5_-carrying *E. coli* belonging to ST648, isolated from a Eurasian otter and a hospitalized patient in Lebanon. We performed whole-genome sequencing on both isolates using short- and long-read sequencing and phylogenetic comparison with publicly available *E. coli* ST648 genomes to assess the genetic relatedness of carbapenem-resistant isolates from other geographic regions.

## 2. Materials and Methods

### 2.1. Bacterial Isolation and Antibiotic Susceptibility Testing

As part of a wildlife surveillance program conducted by the Lebanese Wildlife Team, a carbapenem-resistant *E. coli* (CREC) isolate, designated ECOL198, was recovered from a Eurasian otter (*Lutra lutra*). The otter was alive and wild-caught, and fresh fecal samples (<24 h) were collected aseptically in sterile 15 mL Falcon tubes (Corning^®^, New York, NY, USA). In parallel, another CREC isolate, ECOL247, was obtained during routine clinical testing at the bacteriology laboratories of the American University of Beirut Medical Center (AUB-MC) (Beirut, Lebanon). For further genomic study, ECOL247 was received and transported aseptically under refrigeration conditions (4 °C) at the Experimental Pathology, Immunology, and Microbiology Research Laboratory at the American University of Beirut. Both isolates were identified and confirmed using API 20E strips (bioMérieux, Marcy-l’Étoile, France). Antimicrobial susceptibility testing was performed using the broth microdilution (BMD) against 14 different antimicrobials from 10 different antimicrobial classes: amikacin, aztreonam, trimethoprim-sulfamethoxazole, cefepime, ceftazidime, cefuroxime, colistin, ertapenem, gentamicin, levofloxacin, meropenem, piperacillin/tazobactam, tetracycline, and ceftolozan/tazobactam. Commercially available 96-well microtiter plates (Corning 3788) were used for all broth microdilution assays. The control strain *Escherichia coli* ATCC^®^ 25922 was used in parallel. The minimum inhibitory concentration (MIC) values were assessed according to the clinical breakpoints listed in the Clinical and Laboratory Standards Institute (CLSI) document M100Ed35 [19].

### 2.2. Whole-Genome Sequencing and Analysis

Genomic DNA was obtained using the Quick-DNA™ Fungal/Bacterial Miniprep kit, and then DNA was purified using the Genomic DNA Clean & Concentrator™ kit (Zymo Research, Irvine, CA, USA). Short-read whole-genome sequencing was carried out using the Illumina MiSeq platform (Illumina, San Diego, CA, USA).

Long-read whole-genome sequencing was performed using the Oxford Nanopore MinION Mk1C platform (Oxford Nanopore Technologies, Oxford, UK). Genomic DNA libraries were prepared with the Rapid Barcoding Kit 96 (SQK-RBK110.96) (Oxford Nanopore Technologies, Oxford, UK) and sequenced on R9.4.1 flow cells (FLO-MIN106) (Oxford Nanopore Technologies, Oxford, UK) following the manufacturer’s protocols. Raw sequencing data were processed using tools available on the UseGalaxy platform (https://usegalaxy.org/, accessed on 10 January 2024). Plasmid replicons with a cutoff of above 90% identity, multi-locus sequence typing (MLST), serotypes, phylogroups, pathogenicity, and virulence factors were identified using tools from the Center for Genomic Epidemiology (http://www.genomicepidemiology.org/, accessed on 24 January 2024) [20]. Antimicrobial resistance genes were detected using the CARD (https://card.mcmaster.ca/, accessed on 22 May 2024) and AMRFinder databases (version 3.12.8, accessed on 22 May 2024) [21].

### 2.3. Comparative Analyses of ST648 and bla_NDM-5_-Harboring Genomes

*E. coli* ST648 genomes and corresponding metadata were retrieved from the NCBI Genbank database. Both isolates analyzed in this study were compared with 30 previously sequenced *E. coli* genomes (Table A1). Whole-genome MLST-based phylogeny was conducted using the canu-wgMLST command-line pipeline (https://github.com/marbl/canu.git, accessed on 20 May 2024) (PMID: 31396192). The resulting Newick-format tree was visualized using the R packages ggplot2 (https://ggplot2.tidyverse.org, accessed on 22 May 2024) and ggtree (version 4.0.1, accessed on 22 May 2024) (PMID: 38867905).

### 2.4. Data Availability

The samples analyzed include BioProject PRJNA613441 with BioSamples SAMN44683810 (ECOL198), SAMN53307536 (ECOL198_Long), SAMN53235178 (ECOL247), and SAMN53307537 (ECOL247_Long). Accession numbers for the remaining samples in the phylogenetic tree are provided in Appendix A.

## 3. Results

### 3.1. Basic Genomic Features

In Silico genotyping revealed that both *E. coli* isolates, ECOL198 and ECOL247, belonged to ST648, phylogroup F, and O9:H4 serotype. PathogenFinder indicated both isolates as a potential human pathogen with a probability of 93.2%. Analysis of the sequences showed that the two isolates have comparable genomic properties, including genome size, GC content, and the number of contigs (Table 1). Further, plasmid replicons IncFIA, IncFIB (AP001918), and IncFII (pRSB107) were detected in both isolates. In addition, ECOL247 also harbored the Col (MG828) plasmid (Table 2).

### 3.2. Phenotype of E. coli Isolates

The detailed antimicrobial resistance profiles of both isolates are listed in Table 3. Both ECOL198 and ECOL247 exhibit broad multidrug resistance, with resistance to most β-lactams—including extended-spectrum cephalosporins, β-lactam/β-lactamase inhibitor combinations, monobactams, and carbapenems. They are also resistant to fluoroquinolones, sulfonamides, and tetracycline. They are only susceptible to colistin and amikacin, although gentamicin resistance persists in both strains. Overall, the profiles demonstrate a carbapenem-resistant, multidrug-resistant (MDR) phenotype.

### 3.3. Antimicrobial Resistance Genes and Virulence Genes

**Figure 1:**
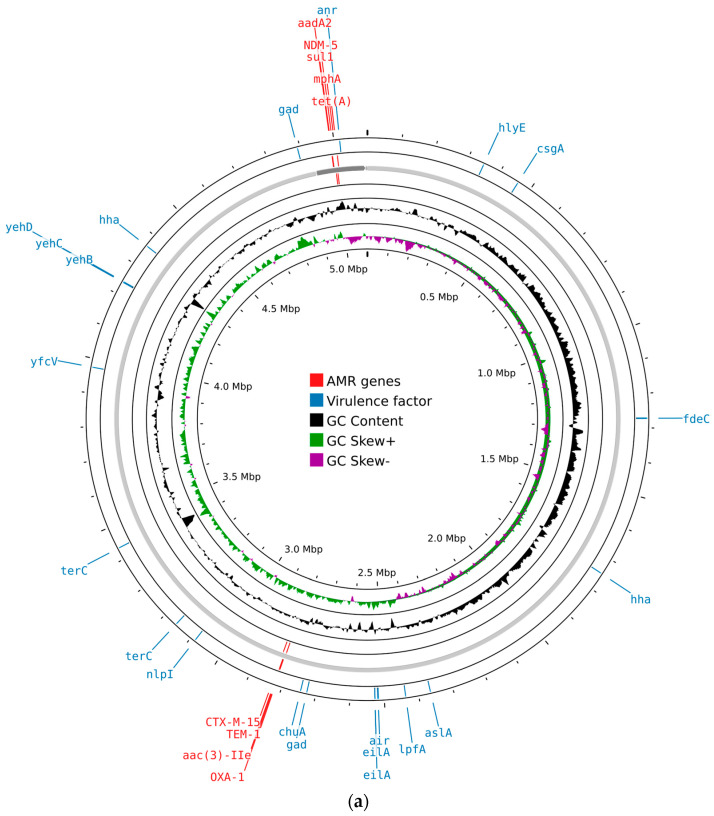

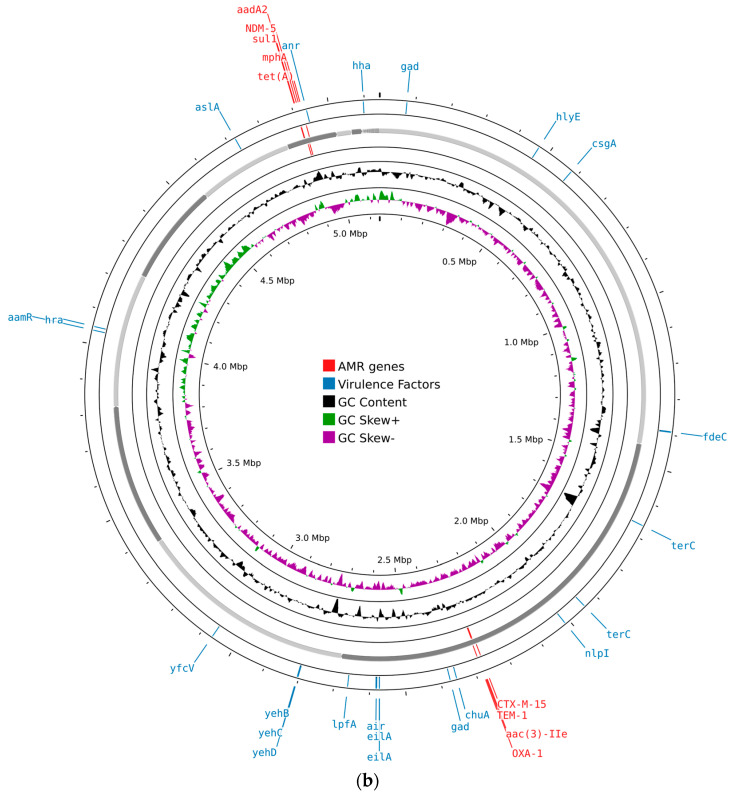
Circular maps of the genomic features of *E. coli* isolates generated using Proksee (https://proksee.ca/, accessed on 22 May 2024): (**a**) circular map of ECOL198; (**b**) circular map of ECOL247. Different features are shown in different colored bars, with virulence factors in blue and resistance genes in red.

AMRFinder-based screening identified the carbapenemase gene *bla*_NDM-5_ and β-lactamase genes (*bla*_TEM-1_, *bla*_CTX-M-15_, and *bla*_OXA-1_), fluoroquinolone resistance-associated mutations in the genes *gyrA* and *parC*, a macrolide resistance gene (*mphA*), aminoglycoside resistance genes (*acc(3)-lle*, *aadA2*), a sulphonamide resistance gene (*sul1*), and a tetracycline resistance gene (*tet*(A)) (Figure 1).

Both *E. coli* isolates carried a range of virulence-associated genes. These included genes related to adhesion and invasion (*aslA*, *chuA*, *csgA*, *fdeC*, *lpfA*, and *yfcV*), iron uptake (*chuA*), and bacteriocin production (*hlyE*). Genes involved in protection against environmental stress and host defenses were also present, such as *gad*, *hha*, *nlpI*, and *terC*. In addition, genes responsible for capsule synthesis (*yehB*, *yehC*, and *yehD*) were detected. Other virulence factors included *air*, *anr*, and *eilA*, contributing to the overall pathogenic potential of the isolates (Figure 1).

### 3.4. Comparative Analysis of the E. coli Isolates

**Figure 2:**
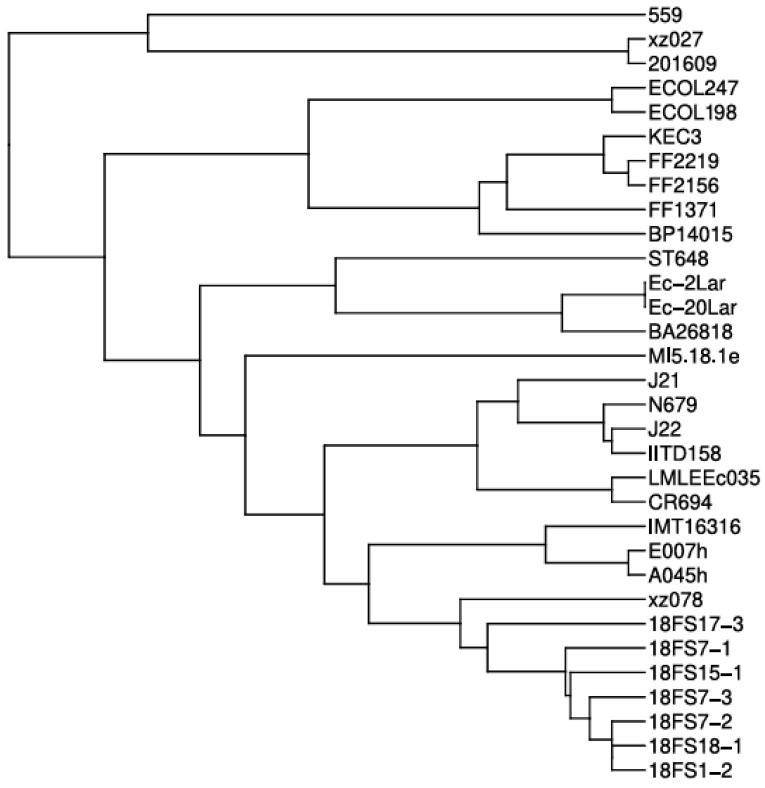
Whole-genome multilocus sequence typing (wgMLST)-based phylogenetic tree of *Escherichia coli* ST648 isolates carrying *bla*_NDM-5_. The tree shows the evolutionary position of our isolates among globally distributed ST648 strains.

To determine the phylogenetic relationship of both *E. coli* ST648 isolates with previously characterized global isolates carrying *bla*_NDM-5_, we performed whole-genome multilocus sequence typing (wgMLST) analysis (Figure 2). The results revealed that ECOL198 and ECOL247 clustered tightly together, suggesting a recent common ancestor and indicating a near-clonal relationship. These two isolates formed a distinct sub-clade with BP14015, a strain isolated from a patient’s blood in India, which branched off earlier in the tree. Notably, this clade also showed phylogenetic proximity to FF isolates (FF2137, FF2156, and FF2219) that were isolated from human clinical samples. Overall, our isolates formed a clearly separated clade, distinct from most publicly available global isolates, highlighting their unique phylogenetic position.

## 4. Discussion

The emergence of carbapenem-resistant *E. coli*, particularly high-risk clones such as ST648, poses a significant threat to public health. Worldwide, several studies have reported the presence of *bla*_NDM-5_-producing ST648 isolates in both clinical and non-clinical settings [22]. This study provided genomic and phenotypic features of *bla*_NDM-5_-carrying isolates, ECOL198 from a wild Eurasian otter and ECOL247 from a hospitalized patient in Lebanon. In addition, we evaluated the phylogenetic relationships of *E. coli* ST648 isolates from diverse sources, retrieved from public databases with sequenced draft genomes of our two isolates.

Interestingly, both isolates exhibited a high degree of genomic similarity: they belonged to phylogroup F and carried an identical set of beta-lactamase genes (*bla*_NDM-5_, *bla*_TEM-1_, *bla*_CTX-M-15_, and *bla*_OXA-1_). Both isolates harbored IncF plasmid types (IncFIA, IncFIB, and IncFII), which are known vehicles for horizontal gene transfer of AMR and virulence determinants. Studies have shown that *E. coli* ST648 is an emerging MDR, high-risk clonal lineage that is regularly detected in wild bird populations, waterfowl, companion animals, and humans [23]. Our detection of resistant *E. coli* ST648 in clinical and wildlife samples supports its classification as a host generalist rather than a specialist, capable of frequent cross-species transmission, thriving in various clinical and nonclinical settings [5]. Of particular concern is the detection of such clinically relevant multidrug-resistant bacteria in wildlife, which are typically not exposed to direct antibiotic pressure. This phenomenon is likely driven by increased human population and habitat fragmentation that forces humans and wild animals to be in closer proximity [24], or it could be due to environmental contamination, as water polluted with feces appears to be the most significant vector of contamination [25]. Overall, the presence of potentially pathogenic *E. coli* lineages such as ST648 in wildlife raises concerns about wildlife serving as reservoirs or spillover sources for high-risk *E. coli* clones. For example, an outbreak of *E. coli* infection resulting in 15 illnesses and two deaths was traced back to Oregon-grown strawberries contaminated with wild deer feces [26]. In another case, eight children contracted *E. coli* following exposure to wild Rocky Mountain elk feces on a soccer field in Colorado [27].

Additionally, PathogenFinder predicted both *E. coli* ST648 isolates to be potential human pathogens with a high probability score of 93.2%. This computational prediction aligns with the presence of several hallmark ExPEC-associated virulence genes in both genomes, including *chuA*, *fdeC*, *yfcV*, and *hlyE*, which are well-documented mediators of iron acquisition, adhesion, and toxin production in extraintestinal pathogenic *E. coli*. Among ExPEC, UPEC are defined as strains positive to two or more *chuA* loci, *fyuA*, *vat*, and *yfcV* [28]. According to these criteria, both *E. coli* ST648 strains studied likely have the potential to be uropathogens, as they carried both *chuA* and *yfcV*. Added to this, these isolates belong to phylogroup F, which is closely related to phylogroup B2 that includes the majority of strains associated with UTIs [3]. Moreover, the high pathogenicity score suggests that these isolates possess a genomic backbone compatible with human infection, even though one was derived from a wildlife source. This finding parallels previous findings demonstrating that wild animal species may be carriers of clinically important resistance and virulence determinants [29]. Similar findings have been reported in other studies where *E. coli* ST648 strains from environmental or animal origins displayed virulence and resistance profiles characteristic of human pathogens [23]. Notably, these results underscore the zoonotic potential of wildlife-associated ST648 strains and support the possibility of clonal dissemination and shared environmental exposure sources. It also highlights the relevance of a One Health surveillance approach to monitoring high-risk lineages circulating across environmental, animal, and human interfaces.

Whole-genome phylogenetic analysis demonstrated a near-clonal relationship between our two isolates, despite their origin from different hosts. They clustered together in a distinct subclade alongside a clinical isolate from India (BP14015), suggesting recent common ancestry and possibly regional dissemination of a unique ST648 lineage. Importantly, this subclade was phylogenetically distant from most other ST648 strains harboring *bla*_NDM-5_ present in public databases, indicating limited relatedness to global clones and pointing to a potentially localized evolutionary path.

## 5. Conclusions

The occurrence of carbapenem-resistant *E. coli* ST648 in human and non-human sources represents a growing threat. Wildlife serves as a significant reservoir, facilitating the transmission of resistance to humans and threatening public health. To effectively address this issue, it is essential to monitor antimicrobial resistance in wildlife through conducting nationwide surveillance programs that would adopt a “One Health” approach, recognizing the interdependence of human, animal, and environmental health. In this context, the multidrug resistance profiles of these isolates and their phylogenetic distinctness from global clones underscore the need for urgent action to monitor and control the spread of high-risk clones in both clinical and non-clinical settings. Urgent actions and public health policies must be implemented by local authorities and municipalities to prevent the spread of high-risk clones into the environment and animal populations. Such interventions may include enforcing strict regulations for the management of clinical waste from both healthcare facilities and households with infected individuals [30], as well as upgrading aging water infrastructure and ensuring that wastewater treatment plants are adequately maintained to effectively reduce microbial burdens, including antibiotic-resistant bacteria [31]. In addition, strengthening biosecurity measures in animal husbandry—such as keeping farm animals separated from wildlife and introducing vaccination programs—can help lower disease rates and reduce the need for antibiotics in animals [32].

## Figures and Tables

**Table 1:** Basic genomic characteristics of the animal and human isolates.

Isolate ID	Genome Size (Mbp)	GC Content (%)	Number of Contigs
ECOL198	5,096,625	50.65	7
ECOL247	5,143,307	50.58	79

**Table 2:** Detailed characteristics of the plasmids identified in *E. coli* isolates, ECOL198 and ECOL247.

Isolate	Replicon Type (s)	Contig	Size (bp)	Resistance Genes	Virulence Factors
ECOL198	IncFIA	34	29,388	-	-
	IncFII (pRSB107)	43	15,261	*tet*(A)	*anr*
	IncFIB (AP001918)	57	4832	-	-
ECOL247	IncFIA	8	157,225	-	-
	IncFIB (AP001918)			-	-
	IncFII (pRSB107)			*tet* (A)	*anr*
	Col (MG828)	19	1549	-	-

(-): none detected.

**Table 3:** The antimicrobial susceptibility profiles of the animal and human isolates to 14 antimicrobials were assessed using the broth microdilution (BMD) method. Minimum inhibitory concentrations (MICs) for each isolate are reported, with R indicating resistance and S indicating susceptibility.

Antimicrobial Class	Antimicrobial	MIC (ECOL198)	MIC (ECOL247)
**Aminoglycosides**	Amikacin	S (4)	S (4)
	Gentamicin	R (64)	R (128)
**Monobactams**	Aztreonam	R (>512)	R (>512)
**Sulfonamides**	Trimethoprim-sulfamethoxazole	R (>512)	R (>512)
**Cephalosporins**	Cefepime	R (128)	R (512)
	Ceftazidime	R (>512)	R (>512)
	Cefuroxime	R (>512)	R (>512)
**Polymyxins**	Colistin	S (0.5)	S (0.5)
**Carbapenems**	Ertapenem	R (512)	R (256)
	Meropenem	R (32)	R (32)
**Fluoroquinolones**	Levofloxacin	R (32)	R (256)
**Penicillin/Beta-lactamase inhibitors**	Piperacillin/tazobactam	R (>512)	R (>512)
**Cephalosporin/Beta-lactamase i nhibitors**	Ceftolozan/tazobactam	R (>512)	R (>512)
**Tetracyclines**	Tetracycline	R (256)	R (256)

## Data Availability

The data presented in this study are openly available in GenBank at NCBI at https://account.ncbi.nlm.nih.gov/?back_url=https%3A%2F%2Fwww.ncbi.nlm.nih.gov%2Fmyncbi%2F, reference number BioProject 613441.

## Abbreviations

The following abbreviations are used in this manuscript:

AMR	Antimicrobial resistance
MDR	Multidrug-resistant
CREC	Carbapenem-resistant *Escherichia coli*
NDM	New Delhi metallo-β-lactamase
wgMLST	Whole-genome multilocus sequence typing

## Appendix A

**Table A1:** *E. coli* ST648 isolates carrying *bla*_NDM-5_ present worldwide.

Continent	Geographic Location	Strain Name	Accession Number
**Asia**	China, Guangdong	18FS17-3	JADDNL010000046.1
		18FS15-1	JADDNO010000044.1
		18FS18-1	JADDNK010000029.1
		18FS7-3	JADDNP010000027.1
		18FS7-2	JADDNQ010000027.1
		18FS7-1	JADDNR010000031.1
		18FS1-2	JADDNX010000048.1
	China, Shandong	559	MOIL01000023.1
		E007h	RYEE01000044.1
		A045h	RYEK01000045.1
	China, Xuzhou	xz027	JAHQLA010000004.1
		xz078	JAHQMY010000031.1
	China, Beijing	strain_ST648	CP008697
	China	201609	CP048107
	Bangladesh	LMLEEc035	JACHQF010000062.1
	India, Delhi	IITD158	JAGJWJ010000056.1
	India, Rajasthan	J21	JADDOA010000102.1
		J22	JADDOB010000076.1
	India, Vellore	BA26818	JACUXU010000057.1
		BP14015	JACUYT010000075.1
		FF1371	JACUYY010000030.1
		FF2156	JACUZC010000070.1
		FF2219	JACUZD010000070.1
	Kenya, Nairobi	KEC3	JABTVQ010000067.1
	Lebanon	ECOL198	SAMN44683810
		ECOL198_Long	SAMN53307536
		ECOL247	SAMN53235178
		ECOL247_Long	SAMN53307537
**Europe**	Germany	IMT16316	CP023815
	Greece	Ec_2Lar	CP035318
		Ec_20Lar	CP035317
	Italy	MI5.18.1e	NZ_JAJSDB000000000.1
	Switzerland	N679	JADDLQ010000002.1
**Oceania**	Australia, Queensland	CR694	JTGI01000069.1
